# “Cultural Security Is an On-Going Journey…” Exploring Views from Staff Members on the Quality and Cultural Security of Services for Aboriginal Families in Western Australia

**DOI:** 10.3390/ijerph17228480

**Published:** 2020-11-16

**Authors:** Lina Gubhaju, Robyn Williams, Jocelyn Jones, David Hamer, Carrington Shepherd, Dan McAullay, Sandra J. Eades, Bridgette McNamara

**Affiliations:** 1 School of Population and Global Health, University of Melbourne, 207 Bouverie Street, Melbourne, VIC 3010, Australia; Robyn.Williams@unimelb.edu.au (R.W.); David.Hamer@nhw.org.au (D.H.); Sandra.Eades@curtin.edu.au (S.J.E.); Bridgette.McNamara@unimelb.edu.au (B.M.); 2 National Drug Research Institute, Curtin University, 7 Parker Place, Technology Park, Bentley, WA 6102, Australia; J.Jones@curtin.edu.au; 3 Telethon Kids Institute, The University of Western Australia, 15 Hospital Avenue, Nedlands, WA 6009, Australia; carrington.shepherd@telethonkids.org.au; 4 Ngangk Yira: Murdoch University Research Centre for Aboriginal Health and Social Equity, 90 South Street, Murdoch, WA 6150, Australia; 5 Kurongkurl Katitjin, Edith Cowan University, 270 Joondalup Drive, Joondalup, WA 6027, Australia; d.mcaullay@ecu.edu.au; 6 Curtin Medical School, Curtin University, 410 Koorliny Way, Bentley, WA 6102, Australia

**Keywords:** cultural security, Aboriginal health, services

## Abstract

Cultural security is a key element of accessible services for Indigenous peoples globally, although few studies have examined this empirically. We explored the scope, reach, quality, and cultural security of health and social services available to Aboriginal and/or Torres Strait Islander families in Western Australia (WA), from the point of view of staff from the services. We recruited staff from health and social services for Aboriginal people in the Perth, Kalgoorlie, Great Southern, and South West regions of WA between December 2015 and September 2017 to complete online surveys. We examined the proportions of participants that responded saying the service was culturally secure, the reasons for the response, and perceived factors related to a high-quality service. Sixty participants from 21 services responded to the survey. Seventy-three percent stated the service was culturally secure; however, only 36% stated that the staff employed at the service had sufficient knowledge on cultural security. Participants suggested having Aboriginal staff and better cultural awareness training as methods to improve cultural security within the service. Participants highlighted that staffing, funding for resources, and patient financial difficulties in accessing care as key areas for quality improvement. Much greater effort is required in improving knowledge through on-going training of staff in the practice of culturally safe care. Organisations must also be required to meet specific standards in cultural safety.

## 1. Introduction

Access to high quality and culturally secure healthcare is a key determinant of health for Aboriginal and/or Torres Strait Islander people (hereafter referred to as “Aboriginal”). Five key elements have been identified to improve the effectiveness of health care for Aboriginal peoples: cultural competence, participation rates, organisational structure, clinical governance and compliance, and availability of services [1]. Lack of cultural security in a health service results in inadequate use of health services and therefore, poorer health outcomes [2].

In Australia, cultural awareness, competency, safety, and security are often used interchangeably with different terms being used in different settings, although they have distinct implications [3,4]. Cultural competency and cultural awareness are related terms which are associated with having the basic knowledge on the cultural issues that impact on Aboriginal people’s health [4] and need to be obtained by health professionals in the local context. As defined by Ramsden and colleagues, cultural awareness is “a beginning step towards understanding that there is difference” [5]. Cultural safety, described by Maori nurses in New Zealand, “is the mechanism which allows the recipient of care to say whether or not the service is safe for them to approach and use” [5]. Curtis et al. (2019) have more recently added that cultural safety requires healthcare professionals and organisations “to examine themselves and the potential impact of their own culture on clinical interactions and healthcare service delivery” [4]. Therefore, cultural safety is focused on the clinical environment rather than the culture of the patient [4]. Going even further, there have been several definitions in the literature for “cultural security” which include: (1) an active, respectful recognition of differing viewpoints on the world with an emphasis on tolerance, understanding, and promotion of effective cross-cultural communication [6], (2) cultural security “seeks to create interactions between health workers and health service users that do ‘not compromise the legitimate cultural rights, views, values and expectations of Aboriginal people’” [7], and (3) “cultural awareness and cultural safety are important foundations for the attainment of cultural security” [3]. All these definitions are suggestive of the more interactive and active process involved in attaining cultural security, which is why we have chosen to focus on cultural security (the highest level) in order to examine how staff at services for Aboriginal people are actively delivering a service that is culturally secure.

Studies have shown that the existence of cultural security within the health system improves health outcomes for Aboriginal neonates. For example, a community-based collaborative approach to shared antenatal care services in Townsville (resulting in increased access to antenatal care and attendance by women) was associated with fewer preterm births [8] and lower perinatal mortality rates [9]. Despite the demonstrated benefits, an audit of antenatal services in Western Australia found that about 65% of antenatal services used by Aboriginal women have not achieved a model of service delivery consistent with the principles of culturally responsive care, with few services incorporating Aboriginal-specific antenatal protocols, maintaining access, or employing Aboriginal health workers. This has been attributed to a lack of awareness about the importance of cultural security [10].

Systemic and institutionalised racism is a consistent feature of culturally insecure environments and has been shown to have detrimental consequences on individual wellbeing. A recent Victorian study found that approximately one-third of Aboriginal survey respondents reported having experienced racism in a health care setting (particularly through communication—e.g., being the target of racist jokes, names, or teasing) [11]. Appropriate staff training and on-going, regular practice of cultural security may mitigate processes that reduce quality of care and resultant psychological distress among Aboriginal peoples.

There are few studies that have comprehensively assessed cultural security within services. In a recent systematic review, only five studies have reported on the impact of cultural competence on effective health service delivery for Australian Aboriginal people [1]. The majority of those studies have been in rural/remote settings and have focused on antenatal/maternal health. Recently published reviews have noted that in order to achieve cultural safety within services, the targets should be the healthcare workforce [12] and systems-level interventions [13]. Therefore, in this study, we examined cultural security from the point of view of service staff with various roles within the organisation; this ensures that they will think about the different elements of cultural security and reflect on their own actions and how they can provide a culturally secure service for Aboriginal people. This study is part of a wider project that our research group is leading which aims to identify the determinants of Aboriginal child health outcomes [14]. Since access to culturally secure health services is an important determinant of Aboriginal child health, we aimed to examine staff perspectives on the scope, reach, quality, and cultural security of services targeted for Aboriginal people of Western Australia.

## 2. Materials and Methods

Aboriginal researchers (S.J.E., J.J., and D.M.) led the design of this study as part of the chief investigators team for the “Defying the Odds” study. We designed the survey in consultation with the “Defying the Odds” Aboriginal Community Reference Group. Aboriginal researchers R.W., J.J., and D.M. led the data collection with health and social services and reviewed and interpreted the results. We received ethical approval for the study from the Western Australian Aboriginal Health Ethics Committee (HREC #609), the WA Department of Health (HREC #2015/30), and the WA Country Health Service Ethics Committee (HREC #2016/17).

Research officers identified health or social services aimed for Aboriginal people using online resources or through community networks. Regions of interest included: the South West, Great Southern, Goldfields, and Perth health regions of WA, as they demonstrated significant variation in maternal and neonatal outcomes for Aboriginal people (findings from secondary analyses of a PhD thesis) [15]. We included primary care, community health, public health, hospitals, specialised health services, and other programmes. Social services for drug and alcohol support, social and emotional wellbeing, and law services were also in-scope. Aboriginal research officers invited Aboriginal and non-Aboriginal staff from 39 organisations to participate from September 2015 to December 2017. Aboriginal research officers visited the service and gave participants an option to either complete the survey online or on tablets.

Through consultation with Aboriginal researchers and practitioners, we designed online surveys with multiple choice and open-ended questions to capture the perspectives from the point of view of people who deliver services to Aboriginal people. The survey covered four major domains: (1) Objectives, (2) Target population, (3) Quality and effectiveness, and (4) Cultural security. Quality and effectiveness of the service focused on: staff characteristics, funding, availability of resources, and patient characteristics. The cultural security questions were informed by Riebel and Walkers’ audit tool on cultural security [10]:

(1) Do you believe that this service is a culturally secure environment for Aboriginal people? If Yes, please select from the following list the reasons (multiple responses were accepted and 14 options were given to choose from). If No, how can cultural security be improved?
(2) Do you believe the staff employed here have sufficient knowledge on cultural security?
(3) Do you think the Aboriginal patients/clients attending this service believe it to be culturally secure?
(4) Is the local Aboriginal community supportive of this service?

For questions 2, 3, and 4, we asked a free text follow-up question to establish the main reason for their answer.

### Data Management and Analyses

We developed surveys on REDCap (Research Data Capture Tool) and exported data to SAS (Version 9.4; SAS Institute Inc., Cary, NC, USA, 2017) for analysis. We calculated overall frequencies for each of the main outcomes of interest stratified by (where applicable): (a) Aboriginal status of the respondent; (b) Geographic location (Perth vs. regional areas); (c) Main type of service provided (General health, Women’s, maternal and child, Family support, Alcohol/Drug, Mental Health, Law); and (d) Governance structure of the service (Government vs. non-Government service; Aboriginal community-controlled health service). The type of service provided was further categorised into: (1) General Health, (2) Family support (including women’s, maternal and child), and (3) Other (Alcohol/Drug, Mental Health, Law).

## 3. Results

### 3.1. Respondent Characteristics

Out of 39 services, 21 participated in the study (54% response rate). We had 1 to 12 respondents from each service, with a total of 60 complete surveys. Most of the participating services were in Perth (52%), followed by Goldfields (29%). Approximately 70% were non-government agencies and about half were Aboriginal community-controlled organisations (ACCOs). Services were most commonly categorised as providing family support services (38%) (Table 1).

Approximately a third of the survey respondents identified as Aboriginal and over 60% had worked within the organisation since the year 2010 (5–7 years; Table 2). The majority were medical professionals (27%) or project officers (27%) (Table 2).

### 3.2. Cultural Security 

A total of 73% of the respondents answered saying that the service was culturally secure and the local Aboriginal community was supportive of the service. However, only 37% of the staff stated that they have sufficient knowledge on cultural security. Only 21% of respondents answered “Yes” to all four questions related to cultural security. Of the respondents that answered saying the service was culturally secure, 40% also stated staff had sufficient knowledge on cultural security.

**Figure 1:**
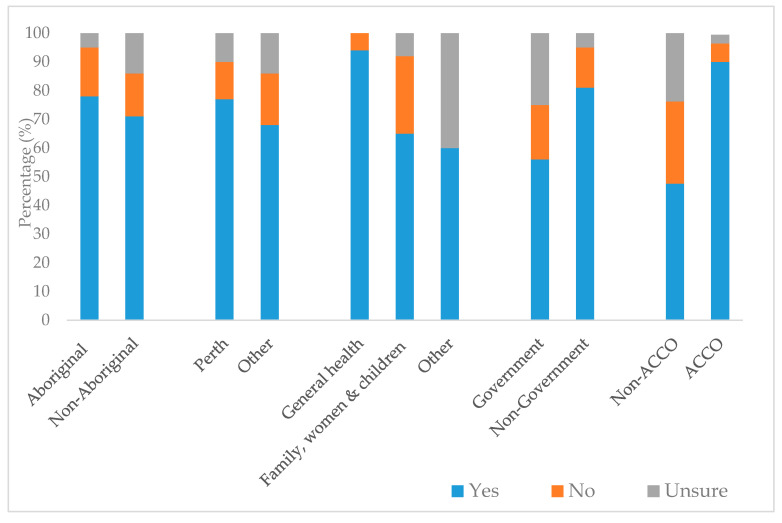
Perceptions of cultural security, by respondent and service-level characteristics.

We found a large difference in perceptions on cultural security among staff at ACCOs compared to non-ACCOs (90% vs. 48% said “Yes”) (Figure 1). A slightly higher proportion of staff located in Perth said the service was culturally secure compared to other regions (77% vs. 68%). Levels of uncertainty about cultural security were highest among non-Aboriginal staff and staff employed at non-ACCOS, government-run organisations, and providing “Other” services.

**Figure 2:**
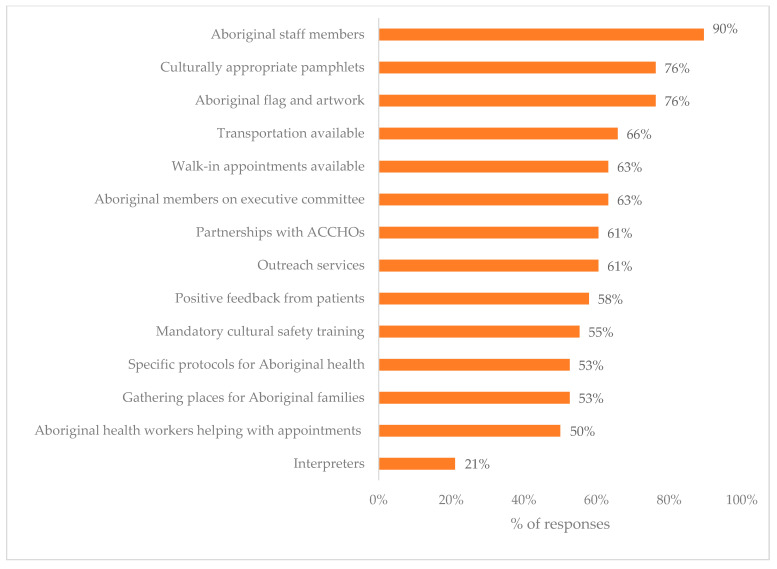
Reasons given from why a service was believed to be culturally secure.

The most commonly reported reasons for cultural security included: Aboriginal staff (90%), Aboriginal flag/artwork displayed (76%), culturally appropriate pamphlets (76%), and transportation (66%) (Figure 2).

Respondents elaborated that there was a general lack of understanding of local issues, a need for more Aboriginal staff, and further implementation of cultural awareness training:

“Staff have technical knowledge but struggle with the context of practice, for example chasing kiddies/families a few times to attend an appt... Understanding what is normal in Aboriginal communities.”

“Cultural security is an ongoing journey and for non-Aboriginal people we can never have enough knowledge.”

### 3.3. Quality and Effectiveness of the Service

We found that over 75% of staff strongly agreed/agreed that the service was: having a positive impact on improving people’s lives, meeting objectives, had happy and satisfied clients, easy to get an appointment at, and recommendable to their family and friends (Table 3). Forty-seven percent responded that the quality of the service required a great deal of improvements. In the stratified analysis, a much higher proportion of respondents strongly agreed that it was easy to get an appointment at: services located outside of Perth (87%) compared to those located in Perth (66%), non-government services compared to government services (83% vs. 56%), and ACCOs compared to non-ACCOs (90% vs. 55%) (Table 3).

We found the main facilitators of an effective service included staff that were: employed long-term, trained, happy/motivated, and good leaders. The primary barrier reported was insufficient number of Aboriginal staff, particularly among government-run services and non-ACCOs (Table 4). Only 8% of respondents agreed/strongly agreed that patients had sufficient money to attend appointments or buy medicine, although this proportion differed by setting—with a higher prevalence among participants in government vs. non-government organisations (19% vs. 3%), Perth metro vs. regional areas (10% vs. 5%), non-ACCOs vs. ACCOs (10% vs. 6%), and general health services vs. services specific to women, children, and families (13% vs. 4%) (Table 4).

## 4. Discussion

This is one of the few studies that has examined the cultural security of health and social services for Aboriginal people as perceived by staff from health and social services. We found that the majority of staff surveyed indicated that the service was culturally secure, mainly due to having Aboriginal staff. Major concerns on the quality of the service that were raised included: inadequate staffing (particularly medical staff, allied health staff, and Aboriginal staff), funding constraints, and patients’ financial situation and ability to manage their health. This is one of the few studies that provides a self-assessment from service providers on the cultural security of the service.

Over 70% of respondents in our study stated that their service was culturally secure. Our results provide a much more favourable view of services for Aboriginal people in WA when compared with a 2010 audit of antenatal services in WA [10], which found that approximately 75% of antenatal services used by Aboriginal women have not achieved a model of culturally safe service delivery. Similar to our study, this audit was undertaken through a telephone survey of representatives from services. However, they recruited only antenatal care services located in rural/remote settings, which may account for the differences in study results. Another evaluation of an antenatal clinic within a tertiary hospital found that the majority (92%) of Aboriginal women felt “mostly understood and respected” by staff, with lower proportions in the birth suite (47%), postnatal ward (31%), and Maternal Fetal Medicine Unit (31%) [16]. We acknowledge that Kildea and colleagues (2012) examined the views from clients, whereas our study was based on the perceptions from service delivery staff, which is likely to account for the differences in findings. We also note that there is likely to be differing views on cultural security from the service provider and client perspectives; in this study, we have chosen to obtain the views from service delivery staff, which demonstrates how staff assess themselves and understand and practice cultural security in the services they work in. To our knowledge, differences in clients and service provider perspectives on the cultural security of the service have not been explored in depth and are an area for future research.

Staff at ACCOs were more likely to report the service as culturally secure. This is consistent with the extant literature, which emphasises the cultural security of such services and the importance that ACCOs have in the health and wellbeing of Aboriginal people [17]. ACCOs have governance models, where each ACCO is not only accountable to, but operated by, the local Aboriginal community. This grounding in local values and culture increases the cultural security of the service. This study supports the existing literature about the importance of cultural security and the role of ACCOs in providing culturally secure care. The finding that staff working at non-ACCOs were about twice as likely to report that the service was not culturally secure highlights the work that needs to be done in non-ACCOs to increase cultural security. If no action is undertaken to improve this, then fewer Aboriginal people will access the services that they need to protect and promote their health.

It was also not surprising that the main reason given for a service to be culturally secure was having Aboriginal staff. This finding accords with the previous literature that shows the importance of health professional characteristics and culture on Aboriginal client’s decisions to access services and also the impact it has on their health outcomes [18]. Munro et al. (2017) [18] describe the importance of “therapeutic alliance” (staff empathy and lived experience) on retention of Aboriginal clients on treatments for drug and alcohol rehabilitation. In contrast, another report suggests that Aboriginality of the staff may not be the key issue; “clients seemed less concerned about the Indigenous status of staff…more important was access to the same provider who was well qualified and experienced, with good listening skills” [16]. The greater uncertainty about cultural security for non-Aboriginal staff also suggests a need for increased and on-going education about the importance of cultural security for all staff [16]; as mentioned by one of the participants, “for non-Aboriginal people we can never have enough knowledge”. In order to successfully practice culturally secure service delivery, both knowledge about cultural security and how it can be implemented at the service level is required. Cultural security training must begin starting at tertiary education settings with on-going face-to-face training for staff led by Aboriginal people and standardised benchmarks for achieving a level of culturally sound care. In this regard, through their scoping review, Jongen and colleagues (2018) [12] have identified that “training and development of the health workforce remain a principle strategy towards the goal of improved cultural competence in health services and systems”. Furthermore, as described by Wilson (2014), it is important that health service staff and people who are undergoing training at all levels (particularly non-Aboriginal people) are taught reflexivity, which will lead to more culturally secure practice at both an individual and organisational level [19].

We identified that staff shortages and insufficient funding were among the major barriers to providing high quality services, which aligns with previous findings of system-wide deficiencies in health services and programs for Aboriginal people, including lack of Aboriginal leadership [20,21]. Our findings also indicate that more support is required for patients in managing their health, appointments, and treatments. Few staff thought patients had sufficient knowledge or skills to follow treatment plans, which suggests a low level of perceived health literacy among patients. Increasing health literacy is important for improving people’s access to healthcare and improving health and wellbeing. A recent study of Aboriginal people in a remote location in northwest Queensland found that young age (<55), gender (female), having only one chronic disease, or having higher levels of education were associated with higher levels of health literacy [22]. To improve health literacy across all demographics, it is important to devise ways of increasing health literacy among Aboriginal people by ensuring it is culturally and traditionally relevant and not focused on Western notions of health and wellbeing.

We found that only a small proportion of staff stated that patients have sufficient money to attend appointments/buy medicine, which reinforces the notion that cost is still a major barrier in accessing healthcare, as previously reported [23]. “Accessibility” best refers to the ability of the health care service or system to respond to the needs of the people [24]. Therefore, further work needs to be done to better support Aboriginal families and implement ways to reduce the cost of healthcare with schemes similar to the Close the Gap PBS Co-payment measure introduced in 2010.

Collectively, the findings from this study underscore the critical need to expand and enhance the cultural security of services for Aboriginal people, and to monitor progress in these areas. The Australian Institute of Health and Welfare’s recently released framework (“Cultural safety in health care: monitoring framework”) will provide a useful tool for assessing progress in this area, showing available data on culturally respectful health care services, Indigenous patient experience of health care, and access to health care services [25].

Strong Aboriginal leadership was a central feature of this project, encompassing study design, data collection, and analyses. Aboriginal research officer connections and networks were essential to successful recruitment. There was a 54% participation rate; the reasons for non-participation included not seeing the relevance of the study for their service priorities and staff having other priorities over research project participation. Future research needs to reach out to these services that we have missed. We acknowledge that we have captured the views and opinions of staff members and the information may be biased and may or may not align with the views of the community that use the services. Seeking the views of the clients of the services is an area of further research that is required to provide a more complete picture of cultural security in these services. Survey questions were based on previous audit tools and multiple questions were asked in relation to cultural security, ensuring that a variety of aspects were captured.

## 5. Conclusions

Significantly more effort is required to ensure practice of culturally safe service delivery to Australian Aboriginal people. All organisations must be required to meet standardized guidelines on cultural security, which includes processes such as continuous and targeted training on cultural security for all staff. More broadly, longer-term processes need to address the relative socioeconomic disadvantage experienced by Aboriginal peoples. These approaches aim to increase Aboriginal people’s access to services to improve Aboriginal population health and wellbeing.

## Figures and Tables

**Table 1:** Characteristics of responding and non-responding services.

	Participating Services (*N* = 21) % (*n*)	Services That Did Not Participate (*N* = 18) % (*n*)
**Location**		
Perth	52 (11)	83 (15)
Goldfields/Esperance/Kalgoorlie	29 (6)	6 (1)
Great Southern	14 (3)	6 (1)
South West	5 (1)	6 (1)
**Gov/Non-Gov**		
Government agency	29 (6)	28 (5)
Non-Government agency	71 (15)	72 (13)
**Aboriginal community-controlled organisation (ACCO)**		
Yes	57 (12)	67 (12)
No	43 (9)	33 (6)
**Main type of services provided**		
General health service (GP)	24 (5)	11 (<3)
Women’s, Maternal, Child health	10 (<3)	22 (4)
Family support	38 (8)	22 (4)
Alcohol/Drug	14 (3)	22 (4)
Mental health	10 (<3)	22 (4)
Legal	5 (<3)	0 (0)

**Table 2:** Characteristics of survey respondents (*N* = 60).

	% (*n*)
**Aboriginal and/or Torres Strait Islander**	
Yes	32 (19)
No	68 (41)
**Age bracket**	
18–24 years	3 (<;3)
25–34 years	14 (8)
35–44 years	27 (16)
45 years or over	54 (32)
**Highest educational qualifications**	
Secondary—Year 12	15 (9)
Diploma—TAFE	20 (12)
Undergraduate/Bachelor’s degree	25 (15)
Post-graduate degree/diploma	38 (23)
**Aboriginal Community Controlled Organisation**	
Yes	53 (31)
No	43 (25)
**Staff start date at service**	
1999 or earlier	15 (9)
2000–2004	7 (4)
2005–2009	15 (9)
2010 onwards	63 (38)
**Position within organisation**	
Medical professional *	27 (16)
Project coordinator/project officer	27 (16)
Support worker/Caregiver ^#^	18 (11)
Executive officer, Manager	13 (8)
Counsellor/Mental health worker ^	7 (4)
Admin officer	3 (<3)

* Aboriginal health worker, community health worker, midwife, registered nurse, specialist; ^ Counsellor, social worker, mental health worker; ^#^ Includes Aboriginal Liaison officer. Due to missing data, the percentages do not always add up to 100%.

**Table 3:** Prevalence of measures of service quality and effectiveness, by service type, location, and governance structure (%).

	Overall	Service Type			Location		Government		Aboriginal Community Controlled	
		General (*n* = 16)	Women, Maternal & Child (*n* = 26)	Other (*n* = 10)	Perth (*n* = 30)	Other (*n* = 23)	No (*n* = 37)	Yes (*n* = 16)	No (*n* = 22)	Yes (*n* = 31)
Objectives being met	75	63	78	90	77	74	78	69	73	77
Quality requires a great deal of improvements	47	63	33	60	40	57	54	31	23	65
Service is having a positive impact	85	75	85	100	80	91	84	88	86	84
People attending are generally happy and satisfied	77	63	78	100	67	91	81	69	77	77
I would recommend this service to my family and friends	83	94	70	100	77	91	87	75	73	90
People find it easy to get an appointment	75	87	59	100	66	87	83	56	55	90

The numbers within the boxes are proportions of respondents that “Strongly agreed/agreed” to the statements shown. A total of 53 respondents answered the questions related to quality and effectiveness of the service. Colour shading is used for visualisation purposes to show differences in proportions within categories and was determined based on the spread of the data with each category (e.g., Service Type, Location, etc.) (Green—highest value; Yellow—median value; Red—lowest value).

**Table 4:** Barriers and facilitators in providing good service by service type, location of service, and governance structure (%).

	Overall	Service Type			Location		Government		Aboriginal Community Controlled	
		General (*n* = 16)	Women, Maternal & Child (*n* = 26)	Other (*n* = 10)	Perth (*n* = 30)	Other (*n* = 23)	No (*n* = 37)	Yes (*n* = 16)	No (*n* = 22)	Yes (*n* = 31)
**Staff Characteristics**										
Adequately trained	65	75	69	40	67	64	69	56	71	61
Good leaders	62	56	69	50	63	59	61	63	71	55
Happy and motivated	46	44	58	20	47	45	50	38	48	45
Number of long-term staff	73	88	73	50	73	73	75	69	71	74
Feel burnt out and overwhelmed	33	38	31	30	37	27	31	38	29	36
**Sufficient staff and resources**										
Medical staff	23	44	12	20	13	36	22	25	14	29
Allied health staff	21	38	12	20	17	27	22	19	19	23
Admin staff	58	63	50	70	47	73	58	56	52	61
Aboriginal staff	33	38	31	30	33	32	33	31	19	42
Funding to improve care	46	63	50	10	57	32	53	31	29	58
Funding for equipment & consumables	33	45	24	38	20	50	35	30	47	24
Space to treat patients	27	44	19	20	23	32	22	38	19	32
**Patient characteristics**										
Attend scheduled appointments	12	13	12	10	13	9	14	6	10	13
Have knowledge/skills to follow treatment plans	17	13	27	0	30	0	14	25	24	13
Have sufficient money for appointments/buy medicine	8	13	4	10	10	5	3	19	10	6

The numbers within the boxes are proportions of respondents that “Strongly agreed/agreed” to the statements shown. A total of 53 respondents answered the questions related to quality and effectiveness of the service. Colour shading is used for visualisation purposes to show differences in proportions within categories and was determined based on the spread of the data (e.g., Service Type, Location, etc.) (Green—highest value; Yellow—median value; Red—lowest value).
